# Theoretical Investigation on Molecular Structure and Electronic Properties of B_x_Li_y_ Cluster for Lithium-Ion Batteries with Quantum ESPRESSO Program

**DOI:** 10.3390/molecules25143266

**Published:** 2020-07-17

**Authors:** Mustafa Ali Çipiloğlu, Ali Özkurt

**Affiliations:** Department of Physics, Faculty of art and Sciences, Manisa Celal Bayar University, TR-145140 Manisa, Turkey; aliozkurt99@gmail.com

**Keywords:** boron-doped lithium, clusters, Ab initio, density functional theory (DFT)

## Abstract

In this study, molecular structure and electronic properties of eleven B_x_Li_y_ (x = 1–3, y = 1–3) clusters are examined using the Perdew, Burke and Ernezerhof (PBE) method in the Quantum ESPRESSO program. Three main groups, consisting of two atoms, three atoms and four atoms, are selected as the starting points. The stable configurations, their binding energies per atom (E_b_), dissociation energy (ΔE), and the second difference in energy (Δ^2^E), HOMO-LUMO (HOMO: Highest Occupied Molecular Orbital LUMO: Lowest Occupied Molecular Orbital) gaps, total energy, frequency, force on atom, point group, bond length, density of state (DOS) and band structures are investigated for B_x_Li_y_ (x = 1–3, y = 1–3) clusters. The results of binding energies (E_b_), dissociation energy (ΔE) and the second difference in energy (Δ^2^E) show that BLi, BLi_2_ first isomer, BLi_2_ second isomer, B_2_Li_2_ first isomer, B_2_Li_2_ second isomer and BLi_3_ are the most stable among all 11 molecules of B_x_Li_y_ (x = 1–3, y = 1–3). The stability of B_x_Li_y_ (x = 1–3, y = 1–3) clusters depend on both the formation of geometrical structures on the number of Li atoms. As the number of Li atoms in the group increases, the stability of B_x_Li_y_ clusters also increases. Within each group formation of geometrical structures, the stability of B_x_Li_y_ clusters changes. It is observed that they may change the capability of chemical reactions in B_x_Li_y_ clusters.

## 1. Introduction

Depletion of fossil energy resources together with severe air pollution are the main reasons why the present world requires a sustainable green energy strategy. Reports of the Intergovernmental Panel on Climate Change clearly showed that a healthy and sustainable environment can only be achieved by supporting clean energy systems. Therefore, experimental and theoretical studies of atomic and molecular clusters have been performed in recent decades for the solution of these problems [1,2,3,4,5,6].

Tailoring of the new materials is a main focus of the researchers. Experimentally, one can tailor new materials from the known empirical data, but most of the researchers carry this out on a trial and error basis. Theoretical simulation is one of the most promising strategies to design and predict new materials and is also a boon for experimental researchers. In view of this, researchers focused on condensed matter physics. Because on this, different physical and chemical properties, atomic and molecular behavior patterns of materials could be actively researched. Nowadays, structures with different geometrical and electronic features are mixed and examined by experimental and theoretical studies [7,8]. Some examples of chemically stable hydrogen storage systems based on boron atom are given in Refs. [9,10]. If we can categorize these new materials, which have more hydrogen storage capability, we can classify eight different groups. These are aluminum nitride nanostructures, transition-metal doped boron nitride systems, alkali-metal-doped benzenoid, Lithium-boron clusters, fullerene clusters, light metal and transition-metal-coated boron Bucky balls, B_80_ and magnesium clusters [11].

Lithium is the lightest metal under normal conditions, and it is a member of alkali metals on the periodic table. It is the best point for a theoretical understanding the physical and chemical properties of metal clusters. Additionally, Lithium can make different heterogeneous clusters with other elements. There are two sub-units of the doped Lithium clusters. The first one is Lithium monoxide (Li_n_O) cluster and the second one is the lithium monocarbide (Li_n_C) cluster [12]. The practical significance of Li-B alloys is as anode materials for the production of lithium batteries. Because of this reason the B-doped lithium clusters have been widely studied. However, contrarily, Li_n_O and Li_n_C clusters are more well-known than B-doped Li clusters [12].

Wang et al. [13] proposed a crystalline structure for Li_5_B_4_ based on X-ray and neutron powder diffraction data, which they confirmed by performing nuclear magnetic resonance measurements. Based on the known atomic (metallic) radii of Li and B, they concluded that the actual Li-Li distances in Li_5_B_4_ are slightly larger than 2.44 Å and the B-B distances are slightly smaller than 1.41 Å. Therefore, Wang et al. observed that the approximate inter-atom distances in Li_5_B_4_ for Li and B are 13–16% shorter than the corresponding inter-atom distances in their metallic state [14]. In the irregular state, they assumed that the distances between atoms within and between four-faced sets are 2.44 Å and 2.67 Å, respectively [14].

Early borides of the alkali metals (NaB_6_) were prepared in 1963 [15]. Additionally, LiB_4_ was claimed in a French patent [16]. However, the existence of LiB_6_ has been suggested [17] and more recently confirmed experimentally [18]. The compounds LiB_2_ and LiB_6_ are reported to have been prepared under pressure at temperatures in excess of 1400 °C [19]. It has also been reported [20,21] that a compound containing about 32% Li exists in the Li-B system. All these Li-B compounds are blackish powders, except LiB_2_ and LiB_6_, which are reported to be golden yellow and bluish black, respectively [19]. All of them are fragile and stable in the air.

From this point of view, Meden et al. computed structures and energetics of the boron-lithium clusters at the SCF/6-31G (d) level [22]. The theoretical investigations on structures, bonding and stabilities of hyperlithiated borides were reported by Nguyen et al. [23,24]. They found BLi_6_ clusters to be most stable among BLi_n_ clusters on the basis of B3LYP cohesive energies of Li and Li_2_ elimination reactions. Bandaru et al. investigated the ability of neutral and cationic B_x_Li_y_ (x = 2–6, y = 1, 2) systems as effective hydrogen-trapping materials at the MP2 level of theory using the 6-311+G (d,p) basis set [11]. Additionally, the lowest energy structures and electronics properties of the BLi_n_ (n = 1–7) clusters were investigated using the B3LYP, MP2 and CCSD (T) methods with the aug-cc-pVDZ basis set [12].

In this study, we obtained the stable configurations of B_x_Li_y_ (x = 1–3, y = 1–3) clusters using the Perdew, Burke and Ernezerhof (PBE) method in the Quantum ESPRESSO program, and used the Chemcraft graphical program for all our clusters and the binding energies per atom (E_b_), dissociation energy (ΔE), the second difference in energy (Δ^2^E), HOMO-LUMO gaps, total energy, frequency, force on atom, point group, bond length, density of state (DOS) and band structures for these B_x_Li_y_ (x = 1–3, y = 1–3) clusters.

## 2. Computational and Mathematical Details

Initially, B_x_Li_y_ (x = 1–3, y = 1–3) clusters have been examined with four methods and two program packages. The Perdew, Burke and Ernezerhof (PBE) method has been studied with the Quantum-ESPRESSO program package. Quantum-ESPRESSO is an abbreviation for Quantum opEn-Source Package for Research in Electronic Structure, Simulation and Optimization. It should be noted that Quantum-ESPRESSO is more efficient for large clusters and extended system wave-function expansions in plane waves [25,26]. We used the Chemcraft graphical program for all our clusters. Chemcraft is a graphical program for working with quantum chemistry computations. It is a convenient tool for visualizing computed results and preparing new jobs for a calculation [27]; moreover, we used BURAI 1.3 for the clusters of Band Structure and DOS graphics [28]. In the computational chemistry, a coupled cluster is a common Post-Hartree-Fock ab-initio method. It expands the multi-electron wavefunction for electron correlation. This method is used for the most correct calculations for small or medium clusters. The PBE functional is a member of the class of generalized gradient approximation (GGA) functional for the exchange correlation energy; PBE developed one example of such a parameter-free GGA functional. It is known for its general applicability and it gives rather accurate results for a wide range of systems [29]. There exist hundreds of the GGA functional. The most famous are the B88 exchange functional and LYP correlation functional and PBE functional [30,31,32].

(1) $$E_{xc}^{GGA}\left[n_{↑},n_{↓}\right]=\int^{\;}d^{3}r\;e_{xc}^{GGA}\left(n_{↑}\left(r\right),n_{↓}\left(r\right),\nabla n_{↑}\left(r\right),\nabla n_{↓}\left(r\right)\right)$$

(2) $$E_{x}^{PBE}=\int^{\;}d^{3}r\;e_{x}^{unif}\left(n\right)\left[1+\kappa -\frac{\kappa }{1+\beta \pi^{2}s^{2}/3\kappa }\right]$$

(3) $$E_{c}^{PBE}=\int^{\;}d^{3}r\;\left[e_{c}^{unif}\left(n\right)+nc_{0}\phi^{3}ln\left\{1+\frac{\left(1+At^{2}\right)\beta t^{2}/c_{0}}{1+At^{2}+A^{2}t^{4}}\right\}\right]$$

(4) $$s\left(r\right)=\frac{|\nabla n\left(r\right)|}{2n\left(r\right)k_{F}\left(r\right)},\;\;\;\;\;t\left(r\right)=\frac{|\nabla n\left(r\right)|}{2n\left(r\right)k_{s}\left(r\right)},\;\;\;k_{s}=\sqrt{4k_{F}/\pi }$$

Binding energies per atom (E_b_), dissociation energy (ΔE), the second difference in energy (Δ^2^E), HOMO-LUMO gaps, total energy, force on atom, point group, bond length (Å), frequency, density of state (DOS) and band structures were calculated for B_x_Li_y_ (x = 1–3, y = 1–3) clusters. The HOMO-LUMO difference energy was also calculated by taking the difference between the HOMO energy and the LUMO energy. A minimal HOMO-LUMO difference means that these nanostructures electrons can pass into LUMO orbitals with smaller electrically potential energy or smaller photon energy. In the optimized clusters the binding energy per atoms formula are computed by using the Equation (5). Lastly, Boron-Lithium clusters were compared. We found that as the number of atoms in clusters decrease, the binding energy per atoms increase due to that the bond making capabilities of boron atoms are greater than Lithium atoms. The dissociation energy means that the energy is needed to break every chemical bond in a molecule and completely separate all atoms. The dissociation energy and the second difference in energy are computed for Boron-Lithium clusters using Equations (6) and (7):

*E (B_x_Li_y_)* = [*x. E(B) + y. E(Li) − E (B_x_Li_y_)*]/*x + y +* 1 (5)

Δ*E (B_x_Li_y_) = E (B_x_Li_y_)* − [*E (B_x_Li_y_) + E (Li)*] (6)

∆*^2^E (B_x_Li_y_)* = [*E (B_x_Li_y+1_) + E (B_x_Li_y-1_)*] − *2E (B_x_Li_y_)* (7)

## 3. Structures and Stabilities of B_x_Li_y_ (x = 1–3, y = 1–3) Clusters

**Figure 1:**
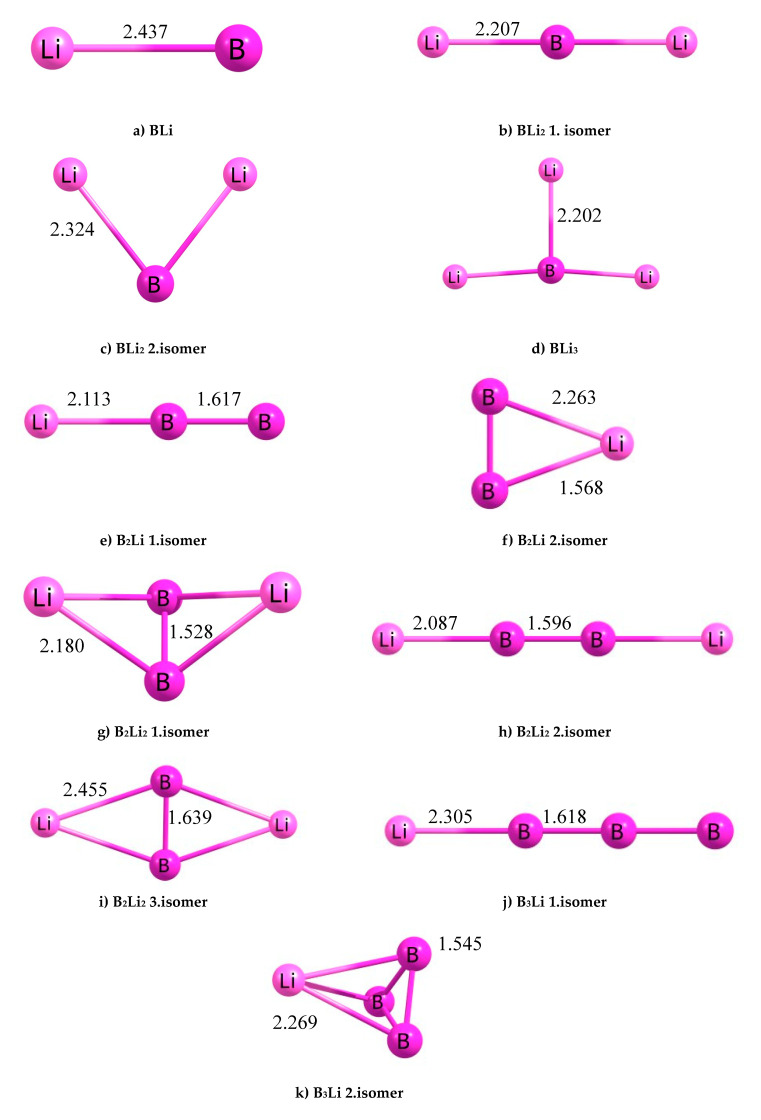
The B_x_Li_y_ (x = 1–3, y = 1–3) molecules.

The B_x_Li_y_ (x = 1–3, y = 1–3) clusters have been selected, with three main groups: two atoms, three atoms and four atoms. We have created 11 different B_x_Li_y_ clusters. The optimized geometries parameters (bond lengths), in accordance with the atom numbers of Figure 1, of the most stable structures are given in Table 1. The lowest energy structures of B_x_Li_y_ (x = 1–3, y = 1–3) clusters were created based off of 11 molecules using PBE.

### 3.1. The B_x_Li_y_ (x = 1–3, y = 1–3) Clusters of Diatomic Group

The BLi molecule is formed by boron and lithium atoms (Figure 1a) The BLi molecule has a very simple geometric form with an average bond length of 2.437 Å. It has C_v_ symmetry. The binding energy per atom, total energy, force on atom and HOMO-LUMO gap information of the BLi molecule are given in Table 2.

### 3.2. The B_x_Li_y_ (x = 1–3, y = 1–3) Clusters of Triple Group

BLi_2_ first isomer, BLi_2_ second isomer, B_2_Li first isomer and B_2_Li second isomer are formed of this group. The initial structure of the BLi_2_ first isomer was made up of two lithium atoms and one boron atom (Figure 1a–c). The BLi_2_ first isomer has a linear geometric form with a 180° angle. The B-Li average bond length is 2.207 Å and Li-Li distance is 4.414 Å. It has D_h_ symmetry. BLi_2_ second isomer is brought forth by two lithium atoms and one boron atom with an angle of 77.84°. This molecule geometry is different from BLi_2_ first isomer. The B-Li average bond length is 2.324 Å and Li-Li distance is 2.921 Å. It has C_2V_ symmetry.

**Figure 2:**
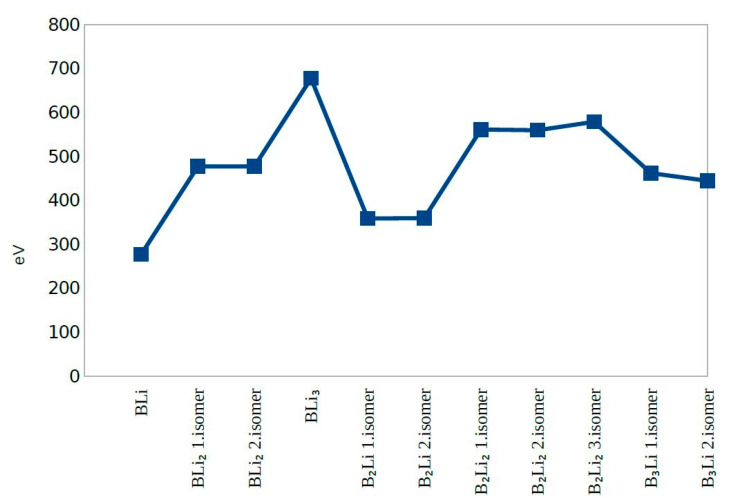
HOMO-LUMO Gap.

**Figure 3:**
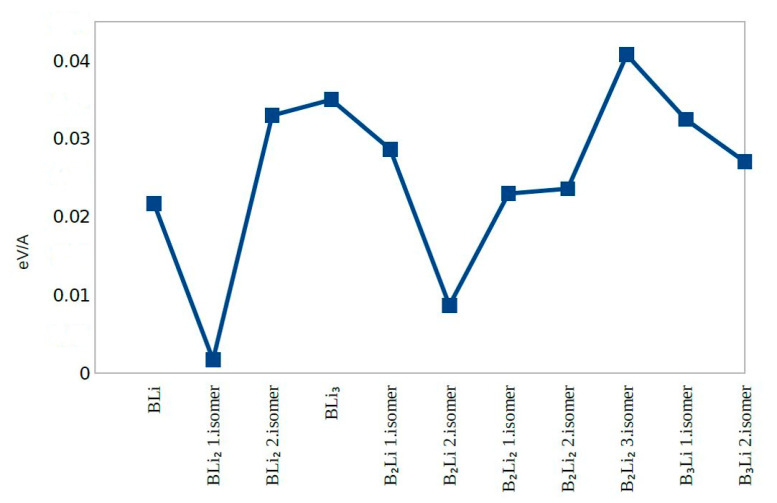
Total Energy.

**Figure 4:**
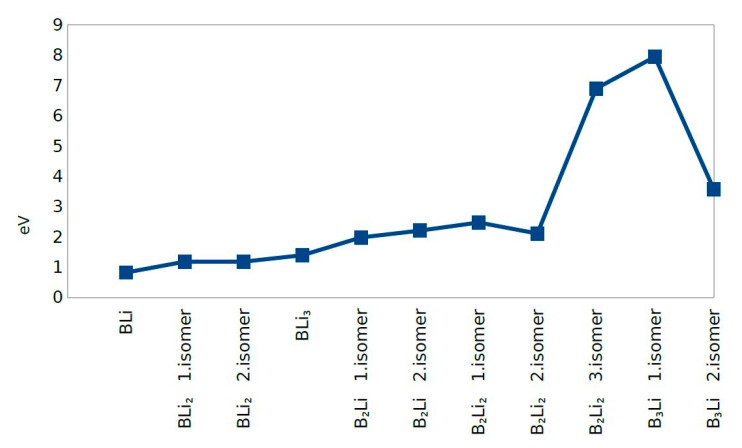
Force on Atom.

B_2_Li first isomer was created with two boron atoms and one lithium atom in Figure 1e,f. It has a linear geometric form with almost a 180° angle and its geometric form is the same form of BLi_2_ first isomer. The B-Li average distance is 2.113 Å and the bond length among boron atoms is 1.617 Å. Symmetry of this molecule is C_S_. Additionally, B_2_Li second isomer is made up of two boron atoms and one lithium atom with a triangle geometric structure, and the angles of the molecule are 69.56°, 69.73° and 40.71°. The B-Li average bond length is 2.263 Å and the B-B average bond length is 1.568 Å. It has C_S_ symmetry. The B_x_Li_y_ (x = 1–3, y = 1–3) clusters of the three atoms group of two molecules are first and second isomers of BLi_2_ molecules and they correspond to a much deeper position on the potential energy surface. This result is also supported by the HOMO-LUMO gap value of these clusters. The HOMO-LUMO energy gap can be used as a measure of chemical hardness, which describes the resistance for a change or deformation of structure [33]. On the other hand, a large HOMO-LUMO energy gap is more stable than a molecule that has a smaller HOMO-LUMO energy gap. For this reason, when we compared four clusters, BLi_2_ first isomer and BLi_2_ second isomer are the most stable molecules within this group. This result has shown in Figure 1; the total energy, force on atom, point group, binding energy per atom, frequency and HOMO-LUMO gap are presented in Table 2 and Figure 1, Figure 2, Figure 3 and Figure 4.

### 3.3. The B_x_Li_y_ (x = 1–3, y = 1–3) Clusters of Quadrate Group

As shown in Figure 1, there are six molecules, which have four atoms among 11 molecules (Figure 1d,g–k). These six molecules are BLi_3_, B_2_Li_2_ first isomer, B_2_Li_2_ second isomer, B_2_Li_2_ third isomer, B_3_Li first isomer and B_3_Li second isomer. BLi_3_ consists of one boron atom and three lithium atoms. The boron atom forms the central atom in this molecule. In addition, three lithium atoms are located in the vicinity of the central atom and are bonded with the boron atom. BLi_3_ is a planar kite-like geometry and can be viewed as a distorted Li_4_ structure, where B impurity substitutes one Li atom. The average bond length of the B-Li cluster is 2.202 Å and the average distance is 3.606 Å among lithium atoms. The angles of BLi_3_ molecule are 178.67°, 89.34° and 98.34°. It has C_1_ symmetry. In this group, BLi_3_ is the molecule with the second smallest HOMO-LUMO energy gap. B_2_Li_2_ first isomer consists of two boron atoms and two lithium atoms. Because of the geometric structure, boron atoms are positioned as central atoms with Lithium atoms at the right side and left side of central atoms. Lithium atoms are located with angled atoms. This molecule consists of four B-Li bonds and one B-B bond. The average B-Li bond length is 2.180 Å, and the B-B bond length is 1.528 Å; lastly, the distance between the two lithium atoms is 3.258 Å. B_2_Li_2_ first isomer has eight different angles (two different 101.99° angles (Li-B-Li), two different 41.29° angles (B-Li-B) and four different 69.35°(angles Li-B-B)). B_2_Li_2_ first isomer has C_1_ symmetry. With regard to HOMO-LUMO energy gap, B_2_Li_2_ first isomer is the second largest of HOMO-LUMO energy gap (Figure 1). This means that B_2_Li_2_ first isomer is the second most stable molecule of this group.

B_2_Li_2_ second isomer has a linear geometric form with two boron atoms and two lithium atoms. Two boron atoms are in the center of the molecule and two lithium atoms are located at the left and right side of the molecule. This molecule formed with three bonds, which are two boron-lithium bonds and one bond of B-B atoms. Additionally, the molecule has a 180° angle. The average B-Li bond length is 2.087 Å and the B-B bond length is 1.596 Å. Additionally, the distance between lithium atoms is 5.770 Å. It has D_h_ symmetry. The HOMO-LUMO gap of B_2_Li_2_ second isomer was calculated as zero. This value is the smallest HOMO-LUMO energy gap in all 11 B_x_Li_y_ clusters. It is understood that the cluster of this molecule will be the most reactive. B_2_Li_2_ third isomer is similar to B_2_Li_2_ first isomer. Two boron atoms were formed the central atoms. One lithium atom was located at the right side and the other lithium atom was located at the left side. All the atoms are in the same plane geometrically. It has four boron-lithium bonds and one boron-boron bond, with a total of five bonds. The average bond length of B-Li cluster is 2.455 Å and the B-B bond length is 1.639 Å. In addition, the distance between the lithium atoms is 4.6298 Å. B_2_Li_2_ third isomer has eight angles (two 55.07° and 57.36° angles (B-Li-B), four 61.00°, 63.93°, 59.64° and 63.00° angles (Li-B-B) and two 120.64° and 126.93° angles (Li-B-Li). The molecule has C_2V_ symmetry group. B_2_Li_2_ third isomer is the third largest of HOMO-LUMO energy gap.

B_3_Li first isomer has a linear geometric structure as BLi_2_ first isomer; for B_2_Li_2_ second isomer, one boron atom is positioned to the left side of the cluster and the lithium atom is positioned to the right side of the cluster. Two other boron atoms are placed linearly between the lithium and boron atoms. The molecule has a 180° band angle, two boron-boron bonds and one boron-lithium bond. Boron-boron average bond length is 2.305 Å and boron-lithium bond length is 1.618 Å. It has C_V_ symmetry. This molecule has the third smallest HOMO-LUMO energy gap. B_3_Li second isomer has a triangle pyramid geometric structure. As a result of this, the molecule has the largest HOMO-LUMO energy gap. B_3_Li second isomer structure has six bonds and 12 bond angles: three boron lithium bond angles of 40.07°, 40.04° and 40.04° (B-Li-B), three boron bond angles of 60.00°, 60.01° and 59.99° (B-B-B) and six boron lithium bond angles consisting of two 69.98° angles (B-B-Li), one 70.11° angle (B-B-Li), one 70.10° angle (Li-B-B), one 69.96° angle (Li-B-B), one 69.86° angle (B-B-Li) and one 69.85° angle (B-B-Li). It has C_1_ symmetry. The B-Li average bond length is 2.269 Å and the B-B average bond length is 1.545 Å.

## 4. Electronic Properties of B_x_Li_y_ (x = 1–3, y = 1–3) Cluster

### 4.1. The Second Difference in Energy (Δ^2^E) and Dissociation Energy (ΔE)

**Figure 5:**
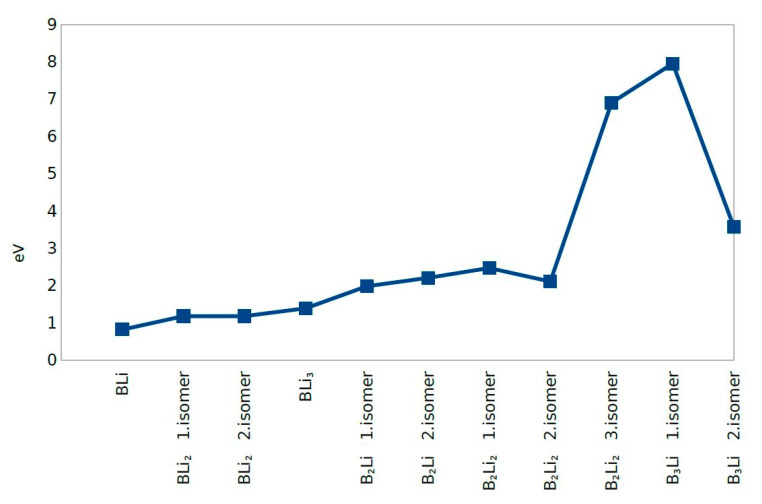
Binding Energy per Atom.

**Figure 6:**
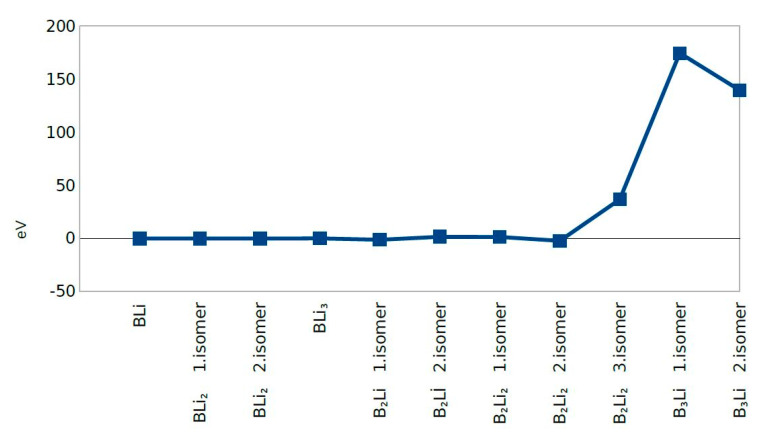
The Second Difference in Energy.

**Figure 7:**
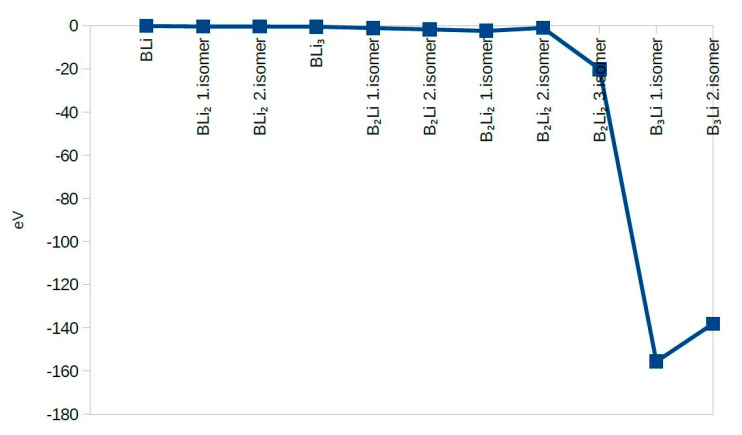
Dissociation Energy.

The second Difference in Energy (Δ^2^E) shows the same value except for B_2_Li_2_ third isomer and B_3_Li first and second isomers. B_3_Li first isomer has the highest value of Δ^2^E among all molecules. The dissociation energy for first eight molecules does not change significantly and dissociation energy values were close to zero. The dissociation energy shows a sharp decrease for B_3_Li first isomer. After combining the second difference in energy and dissociation energy results, BLi, BLi_2_ first isomer, BLi_2_ second isomer, B_2_Li_2_ first isomer, B_2_Li_2_ second isomer, and BLi_3_ actually show the greatest stability among the 11 clusters studied (Figure 5, Figure 6 and Figure 7).

### 4.2. Binding Energy per Atoms

BLi, BLi_2_ first isomer, BLi_2_ second isomer and BLi_3_ have one boron atom in each molecule, while B_2_Li first isomer, B_2_Li second isomer, B_2_Li_2_ first isomer, B_2_Li_2_ second isomer and B_2_Li_2_ third isomer have two boron atoms in each molecule. B_3_Li first isomer and B_3_Li second isomer have three boron atoms in each molecule. The binding energy per atom value is different; B_3_Li first isomer has the highest value and BLi has the smallest value of the 11 molecules for the binding energy per atom. Consequently, the binding energy per atom values is increased with the boron atom number depending on the geometrical structure of each molecule (Figure 5)

### 4.3. Total Energy (eV)

When the total energies of the 11 clusters (which were classified into a diatomic, triple and quaternary atom group) are examined, it is seen that BLi has the smallest total energy in all clusters. In the triple atomic group, B_2_Li first isomer has the smallest total energy and BLi_2_ second isomer has the highest total energy within the group. In the quaternary atomic group, BLi_3_ has the highest total energy and B_3_Li second isomer has the smallest total energy. It can be said that the total energy decreases as the number of Li atom increases, depending on the geometrical shape of molecule. 

### 4.4. HOMO-LUMO Gap

In molecules when light of a high energy is absorbed by an electron in the HOMO, it jumps to the LUMO. For this reason, the energy difference between HOMO and LUMO is termed the HOMO-LUMO gap. HOMO and LUMO are sometimes called frontier orbitals in frontier molecular orbital theory, and we can understand that the difference in energy between these two frontier orbitals can be used to predict the strength and stability of metal complexes. B_2_Li_2_ second isomer has the smallest HOMO-LUMO gap and B_3_Li second isomer has the highest HOMO-LUMO gap. Eventually, the HOMO-LUMO different energies are affected by the geometric structure of molecules and numbers of boron atoms and lithium atoms in the molecules.

### 4.5. Fermi Energy (eV)

The Fermi energy is a notion in quantum mechanics. It generally refers to the energy distinction between the highest occupied and lowest occupied single-particle states in a quantum system of non-interacting fermions at absolute zero temperature. It is changed by the boron atom and the lithium atom, number of molecules and the geometric shape of molecules.

### 4.6. Force on Atom (eV/Å)

The atom consists of three important particles—protons, neutrons and electrons. There are four forces that account for the behavior of the three important particles, and thus, they keep the atom together. The names of these four forces are electromagnetic, gravity, strong and weak interactions. Electromagnetism is a force that amalgamates the effects of electrical charge and magnetism. The electromagnetic force can either attract or repel the particles on which it acts. Opposite-charged particles attract each other, while, same-charged particles repel each other. For example, electrons are kept in the orbit around the nucleus with this force, because the nucleus in the center of the atom is positively charged and attracts the negatively charged electrons. The strong force keeps the protons together to form the nucleus. This goes against the electromagnetic force of repulsion between protons. The atom is held together by both the strong forces and the electromagnetic forces. Weak force is an important force; it stabilizes particles through the process of radioactive decay, in which a neutron in the nucleus changes into a proton and an electron. Inside the nucleus of an atom, the effect of gravity is small compared to the effects of the other three forces. For this reason, gravity is the weakest of the four forces. We can notice that BLi_2_ first isomer has the smallest force value on the atom and B_2_Li_2_ third isomer has the largest force value on the atom. As a result of this information, B_2_Li_2_ third isomer is the most durable molecule. BLi_2_ first isomer is the weakest molecule.

## 5. Results and Discussion

**Figure 8:**
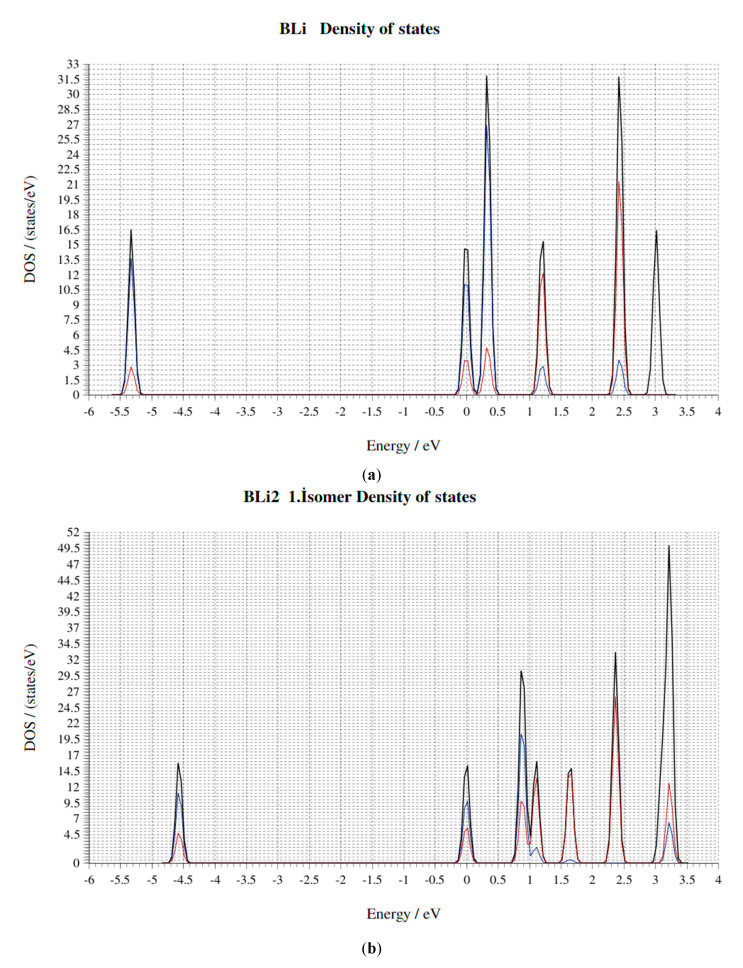

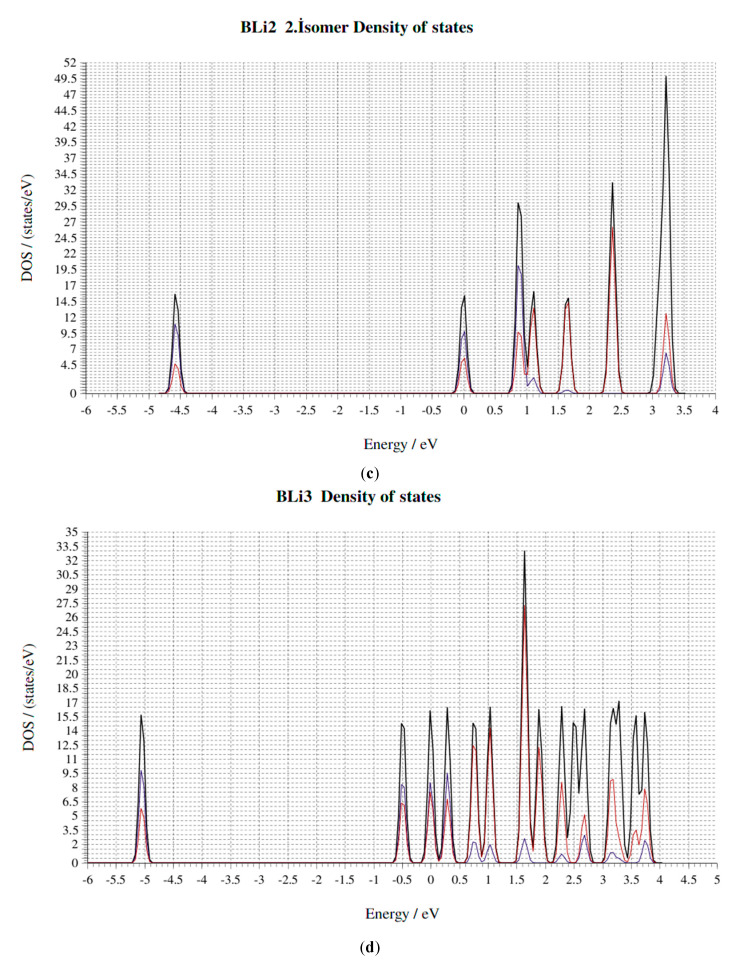

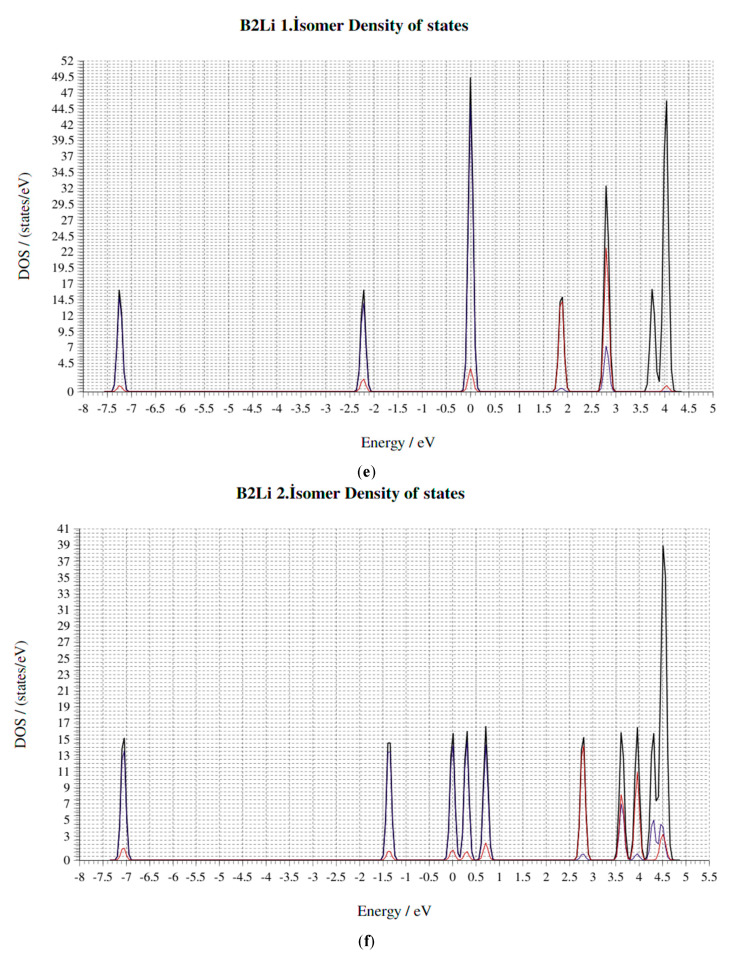

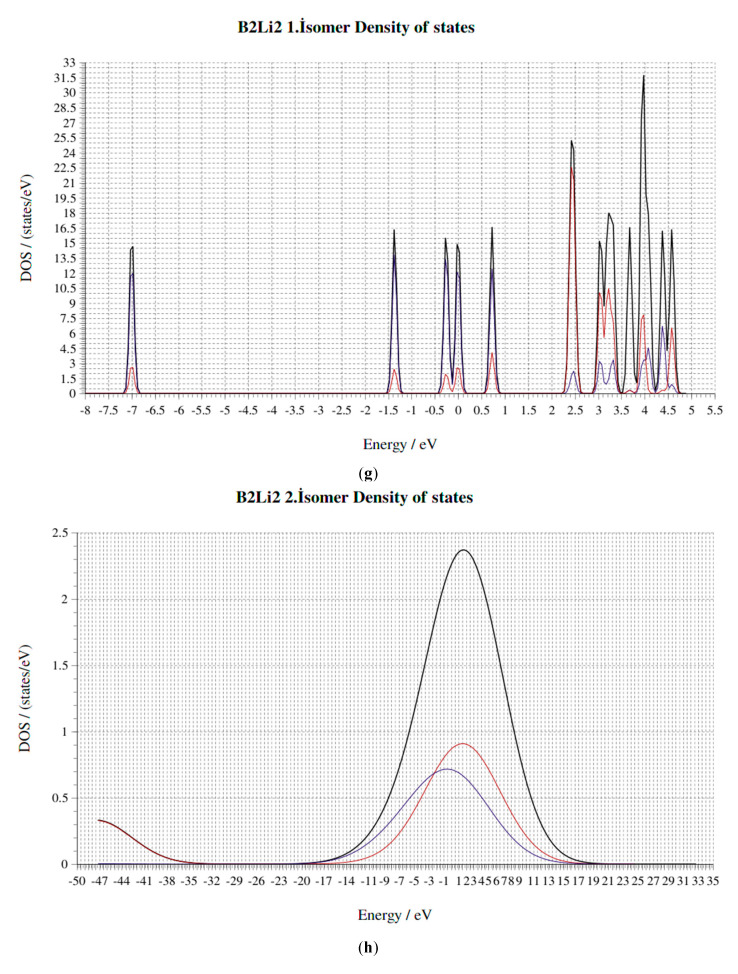

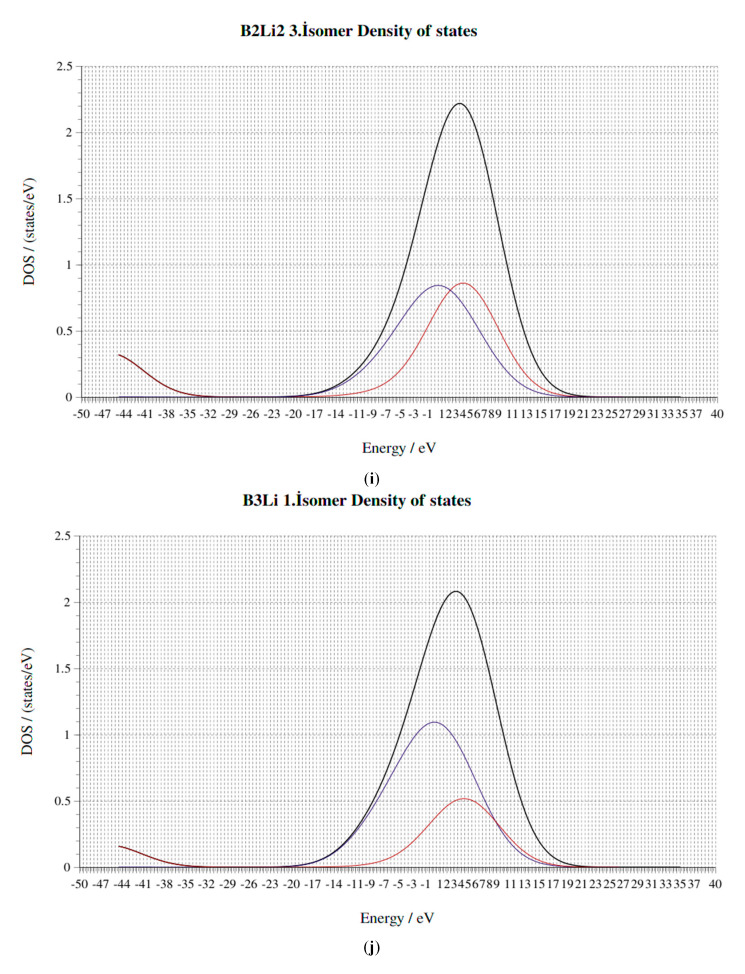

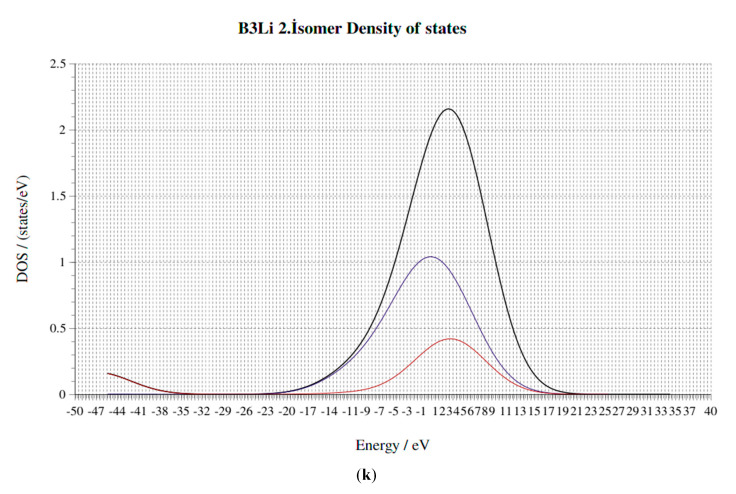
(**a**–**k**). The B_x_Li_y_ (x = 1–3, y = 1–3) clusters of the Density of States.

**Figure 9:**
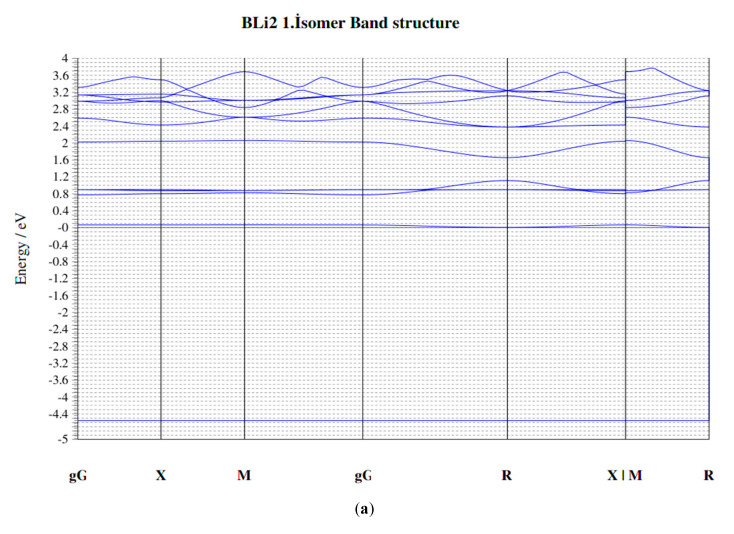

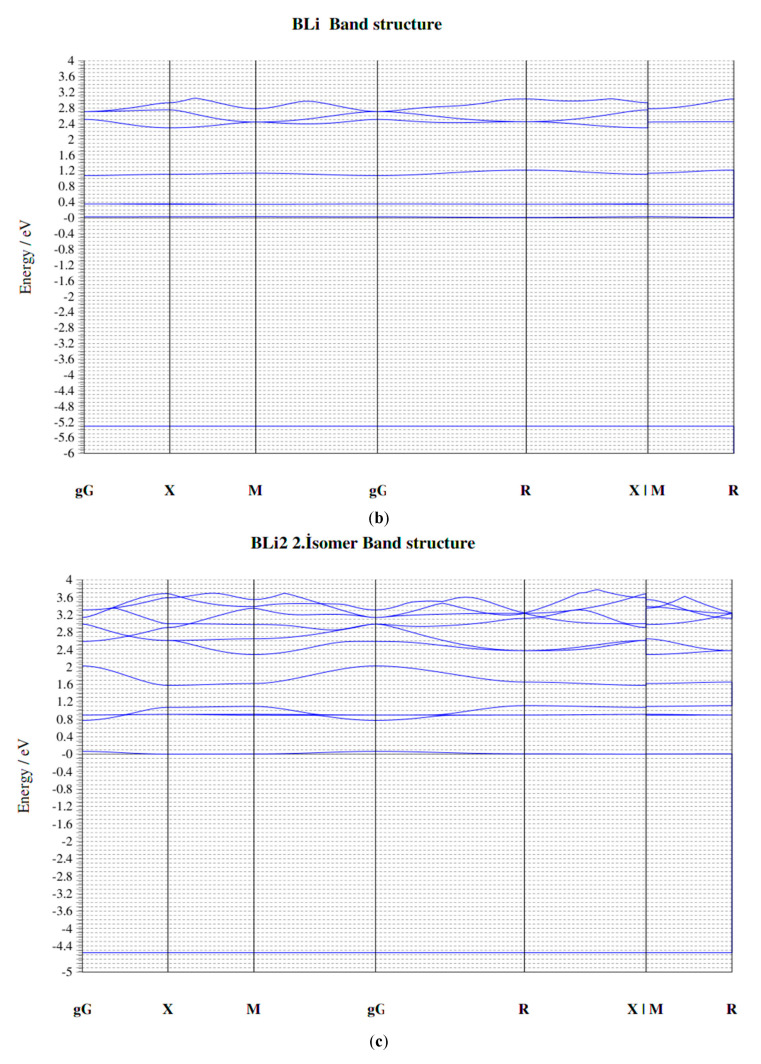

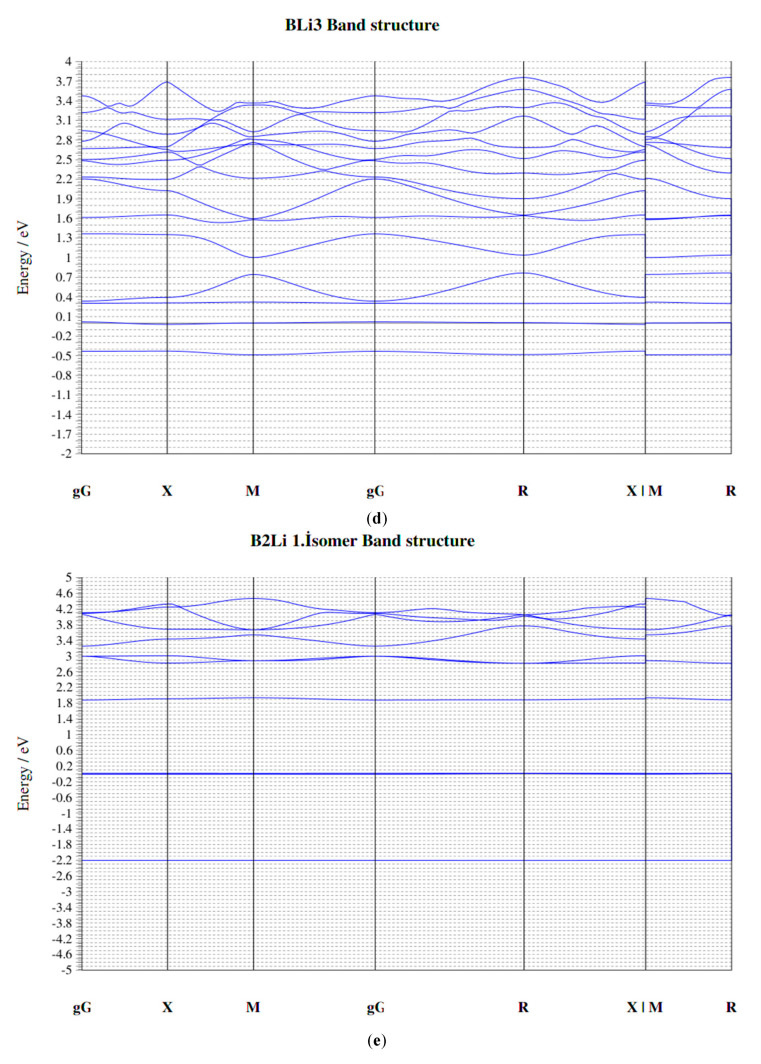

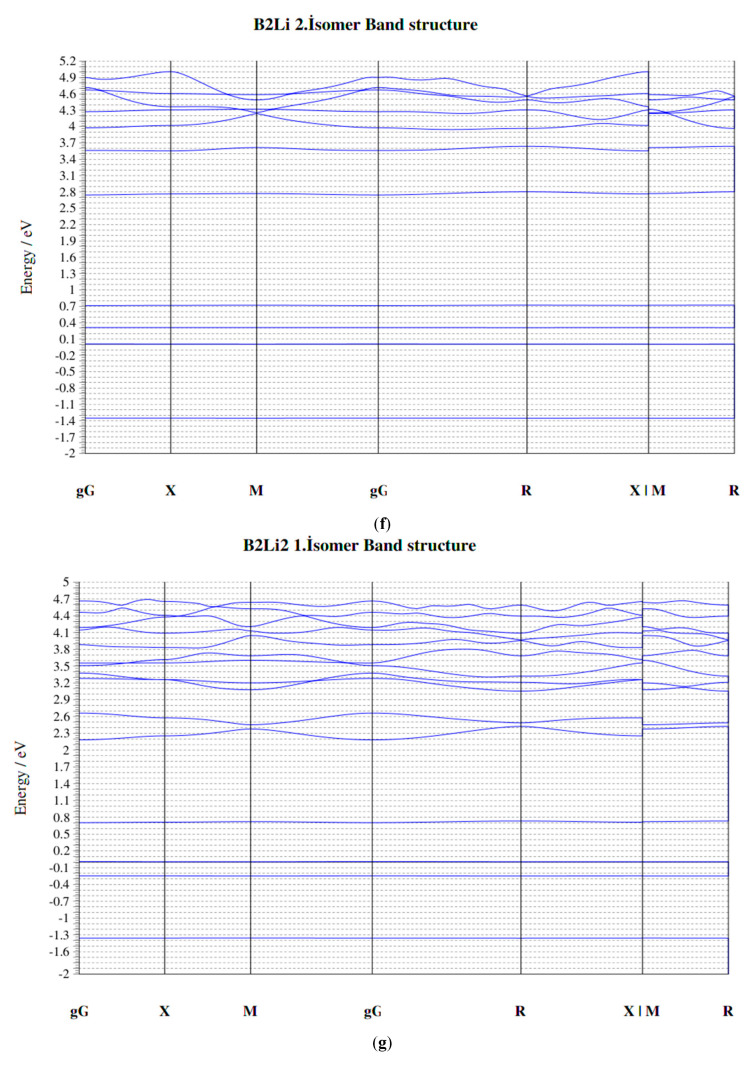

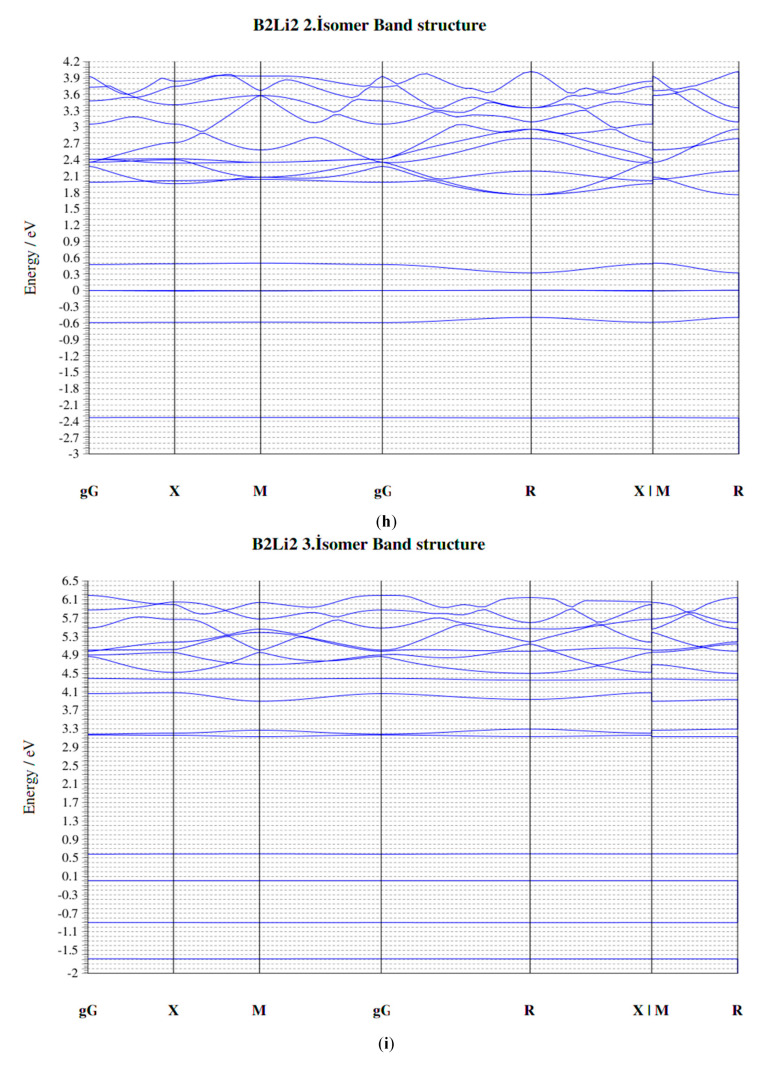

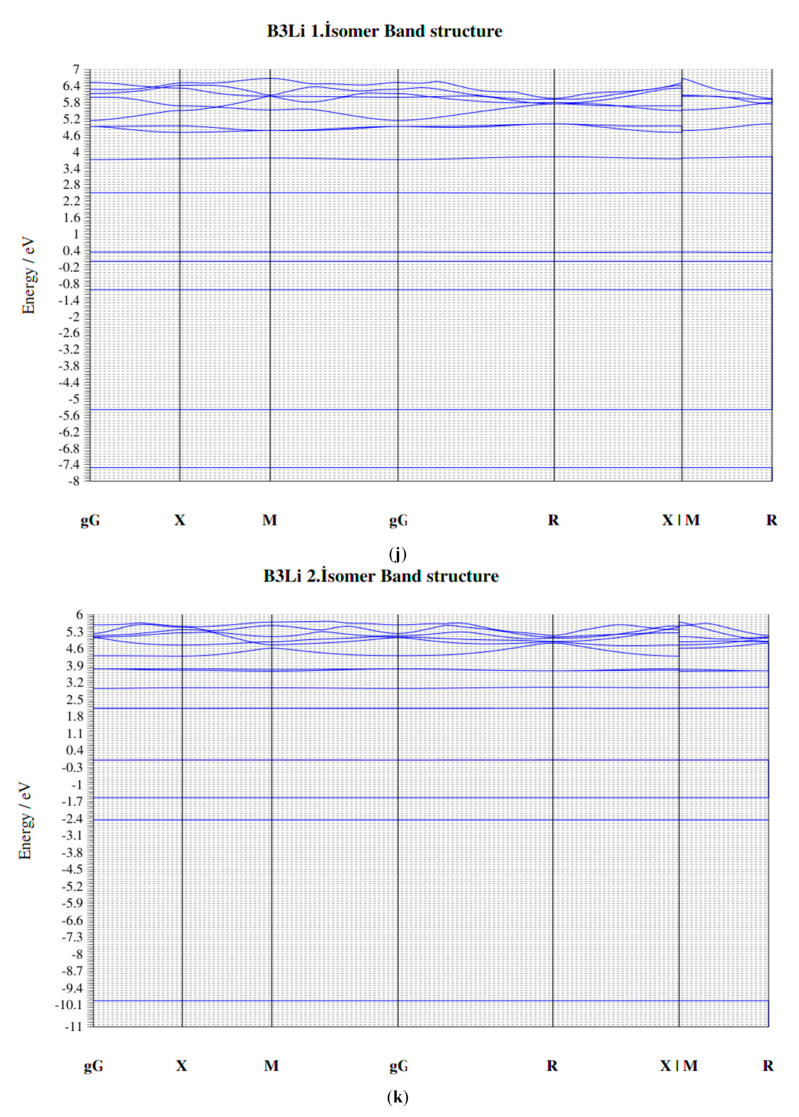
(**a**–**k**). The B_x_Li_y_ (x = 1–3, y = 1–3) clusters of the Band Structure.

The structures and geometrical parameters of the B_x_Li_y_ (x = 1–3, y = 1–3) clusters at the PBE are presented in Figure 1. Total energies, frequencies, the binding energies per atom (Eb), HOMO-LUMO gaps, force on atom, point group and the bond lengths at PBE of all clusters are collected in Table 1 and Table 2. Density of state (DOS) and band structures are collected in Figure 8 and Figure 9. When our results are compared with the well-studied clusters by Nguyen et al. [23], Srinivas et al. [34] and Ying Li et al. [12], we can conclude that our calculations are correct and compatible. The calculated bond length of BLi is 2.141 Å at the QCISD (2.174 Å at the CCSD (T)). The BLi cluster has the largest BLi stretching vibrational frequency of 515 cm^−1^ among all BLi_n_ clusters, indicating a relatively stronger BLi bond than Ying Li et al. [12]. Additionally, the bond length of BLi is 2.416 Å at B3LYP (2.426 Å at UHF, 2.426 Å at MP2 and 2.425 Å at CASSCF) and the BLi stretching vibrational frequency is 425 cm^−1^ [23]. The BLi cluster has a very simple geometric form with an average bond length of 2.437 Å and a BLi stretching vibrational frequency of 428.919 cm^−1^ at PBE in our results. According to Ying Li et al. [12], BLi_2_ second isomer is at the apex, and the Li-Li distance of 2.802 Å is a bit longer than that of the Li dimer (2.727 Å at the CCSD (T)). The B-Li bond length is 2.299 Å at B3LYP (2.323 Å at MP2 and 2.359 Å at CCSD (T)). The B-Li stretching vibrational frequency is 408 cm^−1^. However, the Li-Li bond of BLi_2_ second isomer is 2.779 Å at B3LYP (2.706 Å at UHF, 2.734 Å at MP2 and 2.760 Å at CASSCF). The B-Li bond length is 2.317 Å at B3LYP (2.349 Å at UHF, 2.333 Å at MP2 and 2.365 Å at CASSCF) [23]. The BLi_2_ second isomer stretching vibrational frequency is 430 cm^−1^. BLi_2_ second isomer is made up of two lithium atoms and one boron atom with 77.84° angles at PBE. B-Li average bond length is 2.324 Å and Li-Li distance is 2.921 Å at PBE. The BLi_2_ second isomer stretching vibrational frequency is 420.81 cm^−1^ at PBE in our results. In accordance with Ying Li et al. [12] the Li-Li distance of BLi_3_ is 3.053 Å. The B-Li bond length is 2.158 Å at B3LYP (2.196 Å at MP2 and 2.230 Å at CCSD(T)). The B-Li stretching vibrational frequency is 401 cm^−1^. However, the B-Li bond length is 2.155 Å at B3LYP (2.182 Å at UHF, 2.172 Å at MP2 and 2.199 Å at CASSCF). The BLi_3_ stretching vibrational frequency is 582 cm^−1^, as shown by Nguyen et al. [23]. BLi_3_ has a planar kite-like geometry and can be viewed as a distorted Li_4_ structure, where B impurity substitutes one Li atom. The B-Li average bond length is 2.202 Å and the average distance is 3.606 Å among lithium atoms at PBE. The angles of the BLi_3_ molecule are 178.67°, 89.3° and 98.34° at PBE. The B-Li stretching vibrational frequency is 608.29 cm^−1^ at PBE in our results. The B-B bond distance of B_2_Li is 1.565 Å at B3LYP (1.547 Å at MP2, 1.583 Å at HF and 1.562 Å at CASSCF). In contrast, the B-Li distance of B_2_Li is 2.260 Å at B3LYP, (2.303 Å at MP2, 2.309 Å at HF and 2.294 Å at CASSCF). The B-B bond vibrational stretching frequency is 1118 cm^−1^ for B_2_Li and 1014 cm^−1^ for B_2_ at B3LYP. Total energy of B_2_Li is −56.56928 au at HF (−56.72242 au at MP2, −56.99109 au at B3LYP and −56.72824 au at CASSCF), as shown by Srinivas et al. [34]. B_2_Li second isomer is made up of two boron atoms and one lithium atom with a triangular geometric structure, and the angles of this molecule are 69.56°, 69.73° and 40.71°. The B-Li average bond length is 2.263 Å and the B-B average bond length is 1.568 Å at PBE. The total energy of B_2_Li second isomer is 359.81842 eV at PBE. The B-B bond vibrational stretching frequency is 1112.38 cm^−1^ at PBE in our results. The B-B bond distance of B_2_Li_2_ first isomer is 1.532 Å at B3LYP (1.530 Å at MP2, 1.548 Å at HF and 1.554 Å at CASSCF). Additionally, the B-Li average distances of B_2_Li_2_ first isomer is 2.188 Å at B3LYP (2.217 Å at MP2, 2.252 Å at HF and 2.219 Å at CASSCF). The Li-Li bond distances of B_2_Li_2_ first isomer is 3.352 Å at B3LYP (3.219 Å at MP2, 3.504 Å at HF and 3.382 Å at CASSCF). The total energy of B_2_Li_2_ first isomer is −64.06043 au at HF (−64.25728 au at MP2, −64.57928 au at B3LYP and −64.21198 au at CASSCF), as shown by Srinivas et al. [34]. B_2_Li_2_ first isomer consists of four B-Li bonds and one B-B bond. The B-Li average bond length is 2.180 Å and the B-B bond length is 1.528 Å; the distance between two Lithium atoms is 3.258 Å. B_2_Li_2_ first isomer has eight different angles (two different 101.99° angles (Li-B-Li), two different 41.29° angles (B-Li-B) and four different 69.35° angles (Li-B-B)) at PBE. The total energy of B_2_Li_2_ first isomer is 561.36346 eV at PBE. The B-B bond vibrational stretching frequency is 1170.00 cm^−1^ at PBE in our results. The B-B bond distance of B_2_Li_2_ third isomer is 1.532 Å at B3LYP (1.543 Å at MP2, 1.540 Å at HF and 1.551 Å at CASSCF). The B-Li average distances of B_2_Li_2_ third isomer is 2.156 Å at B3LYP (2.182 Å at MP2, 2.197 Å at HF and 2.177 Å at CASSCF). The total energy of B_2_Li_2_ third isomer is -64.04676 au at HF (−64.27386 au at MP2, −64.57507 au at B3LYP and −64.20757 au at CASSCF), as shown by Srinivas et al. [34]. B_2_Li_2_ third isomer has four boron-lithium bonds and one boron-boron bond, with a total of five bonds. The B-Li average bond length is 2.455 Å and the B-B bond length is 1.639 Å. In addition, the distance between lithium atoms is 4.6298 Å. B_2_Li_2_ third isomer has eight angles (two 55.07° and 57.36° angles (B-Li-B), four 61.00°, 63.93°, 59.64° and 63.00° angles (Li-B-B) and two 120.64°, 126.93° angles (Li-B-Li)) at PBE. The total energy of B_2_Li_2_ third isomer is 559.07518 eV at PBE. The B-B bond vibrational stretching frequency is 1148.02 cm^−1^ at PBE in our results. The B-B bond distance of B_2_Li_2_ second isomer is 1.600 Å at B3LYP and 1.602 Å at MP2, 1.591 Å at HF and 1.560 Å at CASSCF. The B-Li average distances of B_2_Li_2_ second isomer are 2.107 Å at B3LYP, 2.137 Å at MP2, 2.135 Å at HF and 2.142 Å at CASSCF. The total energy of B_2_Li_2_ second isomer is −64.05724 au at HF (−64.20792 au at MP2, −64.54499 au at B3LYP and −64.11602 au at CASSCF), as shown by Srinivas et al. [34]. B_2_Li_2_ second isomer is formed with three bonds, which are two boron-lithium bonds and one bond of B-B atoms. Additionally, the molecule has a 180° angle. The B-Li average bond length is 2.087 Å and the B-B bond length is 1.596 Å. Additionally, the distance between lithium atoms is 5.770 Å at PBE. The total energy of B_2_Li_2_ second isomer is 559.90784 eV at PBE. The B-B bond vibrational stretching frequency is 1171.04 cm^−1^ at PBE in our results.

The boron and lithium atoms make small clusters among them in the B_x_Li_y_ cluster. In particular, Li atoms generally prefer to be close to other Li atoms, unless they are placed symmetrically around the boron clusters, which have shapes similar to the corresponding bare boron clusters. Additionally, depending on the number of lithium atoms and geometrical structure of molecules, an increase or decrease in the average bond length of the small boron clusters are observed. This means that lithium atoms stretch or compact small boron clusters. It appears that this stress also concerns the position of Li atoms and is formed when they are symmetrically located around a small group of boron. Furthermore, the number of Li atoms affects the stability of the cluster, i.e., as the number of Li atoms increases, the total energy of the cluster increases, while the HOMO–LUMO energy gap decreases. These results show that while the stability of the cluster reduces, the probability of the reactivity for the cluster increases with the number of Li atoms. Consequently, we can understand that all molecules have changed the capability of the chemical reaction. The obtained results of the binding energies (E_b_), dissociation energy (ΔE) and the second difference in energy (Δ^2^E) show that BLi, BLi_2_ first isomer, BLi_2_ second isomer, B_2_Li_2_ first isomer, B_2_Li_2_ second isomer and BLi_3_ are the most stable among all 11 molecules of B_x_Li_y_ (x = 1–3, y = 1–3). In addition, the obtained results of the Density of State (DOS) and Band structures show that the LiB clusters do not act as an expectation stability because of highly coordinated boron atoms. It was found that the stability of the molecules in these 11 molecules is changed with the molecule of the boron atoms and the lithium atoms number. Additionally, geometric structures can change the stability of the molecules.

## 6. Conclusions

In summary, the B_x_Li_y_ (x = 1–3, y = 1–3) clusters were examined using the PBE method in the Quantum ESPRESSO program using the Chemcraft graphical program for all our clusters. The stable configurations, their binding energies per atom (E_b_), dissociation energy (ΔE), the second difference in energy (Δ^2^E), HOMO-LUMO gaps, total energy, frequency, force on atom, point group, bond length, density of state (DOS) and band structures were investigated for these B_x_Li_y_ (x = 1–3, y = 1–3) clusters. The obtained results of the binding energies (E_b_), dissociation energy (ΔE) and the second difference in energy (Δ^2^E) show that BLi, BLi_2_ first isomer, BLi_2_ second isomer, B_2_Li_2_ first isomer, B_2_Li_2_ second isomer and BLi_3,_ to be most stable among all 11 molecules of B_x_Li_y_ (x = 1–3, y = 1–3). The stability of B_x_Li_y_ (x = 1–3, y = 1–3) clusters depends on both the formation of geometrical structures and on the number of Li atoms. As the number of Li atoms in the groups increases, the stability of the B_x_Li_y_ clusters also increases. Within each group, the formation of geometrical structures also changes the stability of the B_x_Li_y_ clusters. It is observed that this may change the capability of chemical reactions in the B_x_Li_y_ clusters. Consequently, we can understand that all molecules have changed the capability of the chemical reaction. For these reasons, it can be concluded that using B_x_Li_y_ clusters could be an acceptable strategy to improve the ability of studied nanostructures as new materials of energy storage. Further experimental studies needed to evaluate the potential of B_x_Li_y_ clusters as new materials of energy storage.

## Figures and Tables

**Table 1:** Average Bond Lengths of the B_x_Li_y_ clusters.

Parameters	B–Li	B–B	Li–Li
Bond Lengths (Å)			
BLi	2.437	-	-
BLi_2_ 1. isomer	2.207	-	4.414
BLi_2_ 2. isomer	2.324	-	2.921
BLi_3_	2.202	-	3.606
B_2_Li 1. isomer	2.113	1.617	-
B_2_Li 2. isomer	2.263	1.568	-
B_2_Li_2_ 1. isomer	2.180	1.528	3.258
B_2_Li_2_ 2. isomer	2.087	1.596	5.770
B_2_Li_2_ 3. isomer	2.455	1.639	4.629
B_3_Li 1. isomer	2.305	1.618	-
B_3_Li 2. isomer	2.269	1.545	-

**Table 2:** Properties of the B_x_Li_y_ Clusters.

Clusters/Isomer	Total Energy (eV)	Force on Atom (eV/Å)	Frequency Lowest/Highest (cm^−1^)	Point Group	Binding Energy Per Atom	HOMO-LUMO Gap
BLi	−277.37705	0.0217515	428.919/-	C_V_	0.818640424	0.3392
BLi_2_ 1. isomer	−477.54732	0.0016969	76.22/601.68	D_h_	1.178975414	0.8877
BLi_2_ 2. isomer	−477.54768	0.0329979	214.99/420.81	C_2V_	1.178987446	0.8901
BLi_3_	−677.85093	0.0350184	123.46/608.29	C_1_	1.389984142	0.2944
B_2_Li 1. isomer	−359.13939	0.0286678	164.70/1193.25	C_S_	1.978463931	0.0063
B_2_Li 2. isomer	−359.81842	0.0086903	305.08/1112.38	C_S_	2.204807009	0.3025
B_2_Li_2_ 1. isomer	−561.36346	0.0230113	108.68/1170.00	C_1_	2.470966369	0.7278
B_2_Li_2_ 2. isomer	−559.90784	0.0236314	76.05/1171.04	D_h_	2.107060642	0.0000
B_2_Li_2_ 3. isomer	−579.07518	0.0407777	245.10/1148.02	C_2V_	6.898897329	0.5800
B_3_Li 1. isomer	−462.46380	0.0324987	71.43/1526.35	C_V_	7.948900502	0.3191
B_3_Li 2. isomer	−444.95986	0.0270737	244.13/1245.33	C_1_	3.572915854	2.1249
